# Age-Related Differences in Stepping Response When Stepping onto a Known Soft Surface under Dual Task Conditions

**DOI:** 10.1155/2010/701897

**Published:** 2010-05-26

**Authors:** Nobuko Harada, Shuichi Okada, Shinya Negoro

**Affiliations:** ^1^Graduate School of Human Development and Environment, Kobe University, 3-11 Tsurukabuto, Nada-ku, Kobe 657-8501, Japan; ^2^Hyogo Prefecture Judo Therapists Association, 2-2-25, Tsukamotodori, Hyogo-ku, Kobe 652-0804, Japan

## Abstract

The purpose of this study was to investigate whether age-related differences in stepping response influence postural control when stepping onto a known soft surface under dual task conditions. Nine young and eleven older female adults participated. First, they stepped on a flat surface while grasping an empty cup (single task), and then they repeated the task while grasping a cup filled with water (dual task). For the second experiment, they stepped on a soft surface placed in front of them while performing the above tasks. The main result was that %DIP (initiation phase as a percentage of the total stepping task time) was significantly higher for older than for young adults during the dual task on the soft surface. In conclusion, caution due to previous experience may increase attentional demand during dual tasks and lengthen the time required for central nervous processing in order to avoid losing postural stability in older adults, resulting in reductions in step velocity and step length compared to those in young adults.

## 1. Introduction

The ability to maintain postural stability is a basic requirement for independence and activity in older adults but postural control declines with age, and older adults have difficulty in moving and controlling their posture. Shumway-Cook and Woollacott [1] pointed out that declining balance control might result in increases in the attentional demand associated with maintaining stability. Previous studies have shown that balance ability is reduced when memorizing or counting numbers [2] or performing a math task in older adults [3]. During walking tasks, the attentional demand required for balance is increased by holding a cup filled with water in older fallers [4]. The timed up and go test (TUG) has been used to predict the level of functional mobility, and Lundin-Olsson et al. [5] reported that the TUG is valid for identifying older adults' falling tendency when they are holding a cup of full water. Lajoie et al. [6] investigated age-related differences in the allocation of the attentional resources necessary for sitting, standing, and walking and concluded that standing and walking require greater attentional resources in older adults than sitting. 

One of the factors that cause increases in the level of attentional demand required with age is the need to compensate for sensory system deterioration [7]. To stabilize human posture, there are three sensory systems: the somatosensory, vestibular, and visual systems, and the somatosensory system is particularly important for controlling postural stability because sensory information comes directly from the soles of the feet. However, changes with age in the mechanical properties of the skin and its receptors reduce the afferent information from the soles of the feet that can be used for posture control and cause a reduction in the speed at which older adults move and control over their posture [8]. Previous studies examined whether changes in the stability of postural control or gait could be induced by altering the sensory inputs from the plantar surface with ice intervention [9] or anesthesia [10]. These studies indicated that sensory input from cutaneous feedback in the foot is important in the regulation and modification of gait patterns and the maintenance of normal balance. 

Thus, an age-related decline in the plantar sensory system could result in increases in the attentional demand associated with maintaining postural stability. Teasdale et al. reported that as the amount of sensory information is reduced, postural tasks become more difficult for older adults and require more of their attentional capacity [11]. Shumway-Cook and Woollacott [1] examined the effects of the age and balance ability on attentional demand in various sensory contexts and suggested that in contrast to young adults, in older healthy adults, changes in sensory context, particularly the surface condition, influence the attentional demand required for static postural control. Thus, these studies have verified the significance of sensory context for postural control during dual tasks. However, older adults usually have more difficulty than younger adults in maintaining balance when walking on irregular or uneven surfaces [12]; therefore, it is necessary to examine the effect on attentional demand in dynamic conditions such as stepping or walking when the amount of sensory information from the soles of the feet is reduced.

Lockhart et al. [13] suggested that it is necessary for older adults to make gait modifications, which are called “slip avoidance strategies, before stepping on a known slippery surface in order to avoid falling and requiring an extra step. Interestingly, the older adults reduced their heel contact velocity and required coefficient of friction one step before crossing the contaminated surface, whereas, the young adults adjusted their gait during the condition. Since older adults encounter such situations in their daily life, it is intriguing to assess age-related slip avoidance strategies. Although the authors explored age-related differences in slip avoidance strategies from a biomechanical viewpoint, the changes in attentional demand associated with falling accidents have not been investigated.

In this study, age-related differences in stepping response associated with postural control when stepping on a known soft surface under dual task conditions were investigated. We used a soft surface instead of a slippery surface to ensure that the experiment was safe. Soft surfaces should require more attention because they decrease the afferent information provided from the soles of the feet and so the difficulty of controlling postural stabilization is increased [1]. We hypothesized that older adults would show a different response to stepping on a known soft surface compared to young adults, as they would need to dedicate more attentional demand to the task.

## 2. Methods

### 2.1. Subjects

Nine young (age: 19.0 ± 0.9 years old; mean ± SD ) and eleven older (age: 69.0 ± 3.1 years old; mean ± SD) healthy female adults participated in this study. The young adults were recruited from among Kobe university students. The older adults were volunteers recruited from the community. Each subject gave their informed consent in compliance with the ethical approval granted by the Human Ethics Committee of the Graduate School of Human Development and Environment of Kobe University. 

All adults completed interviews about their musculoskeletal and neurological status, and none had any history of significant musculoskeletal, neurological, or other major systematic medical problems. All adults had an examination for the presence of sensory and motor dysfunction by a physiotherapist and had no physical restriction on their activity.

### 2.2. Experimental Protocol

**Experiment 1**

The subjects were instructed to stand upright and barefoot on a forceplate (Takei Inc., T.K.K 1273a, Tokyo, Japan) and step forward as quickly as possible following a tap cue on the back of their heel provided manually by the experimenter while grasping an empty cup (single task). A sound of tap cue with a rubber hammer was negligible and the sensory of touch of tap cue on the heel triggered the stepping. The stepping task was then repeated with an added manual task. For the performance of stepping alone, they were instructed to stand upright, hold a cup filled with water weighing 200 g, and step as quickly as possible following the tap cue (dual task).

**Experiment 2**

A low-resistance mattress (Yagami Co., YAMF-330G, Tokyo, Japan) half the size of the platform was placed in front of the participants, and they were instructed to put their feet side by side on it moving their right foot first. They were instructed to perform the task in exactly the same manner as in experiment 1, and all subjects performed three trials for each task. To familiarize themselves with stepping onto a soft surface and to demonstrate that they understood the instructions, the subjects performed the test once or twice before they started.

### 2.3. Measurement and Data Analysis

The center of pressure (COP) and ground reaction force data were collected during the stepping task until they had placed their feet side by side, using the forceplate. The data obtained during stepping were sampled at 500 Hz. The forceplate and switch data were synchronized and also analyzed using data analysis software (Flexpro 7.0, Hulinks Inc.).

**Figure 1:**
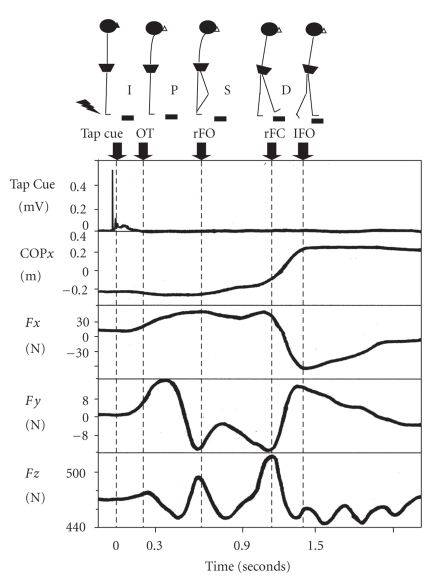
An example of step response data. From top to bottom: Tap cue on back of heel; COP*x* = Mediolateral center of pressure; *F* *x* = Ground reaction force in lateral direction; *F* *y* = Ground reaction force in anteroposterior direction; *F* *z* = Vertical ground reaction force. The stepping task was divided into four phases: (1) the initiation phase (I) was calculated from the tap cue to the onset (OT); (2) the preparatory phase (P) was calculated from OT to foot-off for the right leg (rFO); (3) the swing phase (S) was calculated from rFO to foot-contact for the right leg (rFC); (4) the double-stance stance phase (D) was calculated from rFC to foot-off for the left leg (lFO).

The method for the data analysis of the present study was taken from Brauer et al. [14] and Melzer and Oddsson [15]. The COP*x* (Mediolateral center of pressure) and the ground reaction force data (*F* *x* = ground reaction force in lateral direction; *F* *y* = ground reaction force in anterior-posterior direction; *F* *z* = vertical ground reaction force) have been described in terms of the length of events during the stepping task (Figure 1); onset time (OT) was determined as the first medio-lateral shift of the COP*x* (COP*x* excursion greater than 10 mm away from the baseline following the tap). Time of foot-off for the right limb (rFO) was determined from the trace of *F* *y*, the first lowest peak value at approximately the same time as the trace of *F* *z* and the first highest peak value following OT. The second peak value of the vertical force (*F* *z*) was used to determine the time of foot-contact (rFC), and it was nearly equivalent to the second lowest peak value of *F* *y*. The time of foot-off for left limb (lFO) was determined when the trace of COP*x* reached its final stable value. 

As can be seen in Figure 1, the stepping task is composed of four phases: the initiation phase, the preparation phase, the swing phase, and the double-stance phase: (a) the initiation phase: from the spike of the tap cue to OT, (b) the preparatory phase: from OT to rFO, (c) the swing phase: from rFO to rFC, and (d) the double-stance phase: from rFC to lFO. 

The subjects performed each task three times, and the mean value of the duration of each phase was calculated.

The stepping parameters measured were stride length (cm), step velocity (cm/sec), the lengths of the initiation and preparation phases (sec), and the durations of the initiation phase, the preparation phase, the swing phase, and the double-stance phase as a percentage of the total stepping task time (%).

 A two-way analysis of variance (ANOVA) was used within participants to determine whether stride length (cm), step velocity (cm/sec), the lengths of the initiation and preparation phases (sec), and the durations of the initiation phase (DIP), the preparation phase (DPP), the swing phase (DSP), and the double-stance phase (DDP) as a percentage of the total stepping task time (%DIP, %DPP, %DSP, %DDP) differed significantly between the two age groups. When significant differences were found (*P* < .05), Turkey post-hoc tests were performed.

In addition, executive function was assessed using the modified Trail Making Test (TMT), which was originally developed as part of the Army Individual Test Battery. Coppin et al. [16] showed the validity and reliability that TMT has been widely used in clinical evaluations for the assessment of deficits in executive function. The TMT includes two types, the A and B tests, and both tests are timed [17]. The differences in the scores between the two tests (ΔTMT) were found to be a more accurate measure of executive function than Tests A and B individually [16]. In this study, we converted the English letters into Japanese hiragana and reduced by a half the quantity of numbers and hiragana from the original TMT to produce the modified TMT-A (M TMT-A) and modified TMT-B (M TMT-B) in consideration of the fatigue of the older adults. Moreover, we defined the difference between M TMT-A and M TMT-B as modified ΔTMT (MΔTMT).

The subjects were also assessed using two tests of balance and mobility function. The first was the Berg Balance Test (BBT) [18], which consists of 14 different tasks including the ability to sit, stand, reach, lean over, turn and look over each shoulder, turn in a complete circle, and step. The maximum total score on the BBT is 56, which indicates excellent balance. The other test was the Timed Up and Go Test (TUG) [19], which is timed and useful for predicting the level of functional mobility including the ability to stand up from a chair, walk 3 m as quickly as possible, turn around an object, walk back to the chair, and sit down. 

Unpaired *t*-tests between the older and the young adults were used to determine the effect of age according to the MΔTMT, BBT, and TUG.

All statistical significance levels were set at less than 0.05.

## 3. Results

Table 1 presents the participants' characteristics, and Table 2 presents the parameters of the stepping response under single and dual task conditions for the older and young adults.

### 3.1. M-TMT-A, M-TMT-B, MΔTMT, BBT, and TUG

The detailed characteristics of the participants and their test scores are given in Table 1. The analysis of the M-TMT-A, M-TMT-B, MΔTMT, BBT, and TUG results indicated an influence of age. The mean times of M-TMT-A, M-TMT-B, MΔTMT, and TUG were significantly longer for the older than for the young adults (M-TMT-A: *t* = 7.292, *P* < .001, M-TMT-B: *t* = 5.372, *P* < .001, MΔTMT: *t* = 4.523, *P* < .001, TUG: *t* = 6.347, *P* < .001). The mean BBT score was significantly lower in the older than in the young adults (*t* = 2.825, *P* < .05).

### 3.2. Step Length

Table 2 shows a comparison of the mean step length between the older and young adults. In both tasks, the step length of the older adults was significantly shorter than that of the young adults on both surfaces (flat surface (single: *P* < .001, dual: *P* < .001) and soft surface (single: *P* < .001, dual: *P* < .001)). 

To account for the potential influence of step length, the lengths of the swing and double-stance phases would be inappropriate step parameters when compared between ages; whereas, the lengths of the initiation and preparation phases should not be affected by the step length. Therefore, we determined step velocity, the lengths of the initiation and preparation phases, and %DIP, %DPP, %DSP, and %DDP as step parameters in order to make comparisons between the age groups.

### 3.3. Step Velocity

Table 2 shows a comparison of the mean step velocity during the stepping task between the older and young adults. The step velocity of the older adults was significantly decreased compared to that of the young adults in both tasks on both the flat and soft surfaces. (flat surface (single: *P* < .001, dual: *P* < .001) and soft surface (single: *P* < .001, dual: *P* < .001)).

### 3.4. The Lengths of the Initiation Phase and %DIP

Table 2 shows the mean length of the initiation phase, and Table 3 shows the %DIP during the stepping task in the two age groups on the flat and soft surfaces. The length of the initiation phase varied significantly more in the older adults than in the young adults in both tasks (single: *P* = .001, dual: *P* < .001) on the soft surface, whereas, there were no significant differences on the flat surface.

One interesting result was that %DIP was significantly higher for the older than for the young adults during the dual task on the soft surface (*P* < .05).

### 3.5. %DSP and %DDP

Table 3 shows a comparison of the mean length of each phase as a percentage of the total stepping task time between the two age groups on the flat and soft surfaces. 

Table 3 shows that %DSP was significantly lower for the older than for the young adults during the dual task on the flat surface (*P* = .012) and in both tasks on the soft surface (single: *P* < .05, dual: *P* < .05). %DDP was also significantly higher for the older than for the young adults in both tasks on the soft surface (single: *P* < .01, dual: *P* < .05).

## 4. Discussion

The purpose of this study was to investigate the influence of age-related differences in the stepping response when stepping onto a known soft surface, which worsened their balance, under dual task conditions. 

Previous studies have examined step performance during dual tasks on a firm surface. Melzer and Oddsson [15] found that older adults were significantly slower than young adults in all step parameters under both single and dual task conditions during voluntary stepping. The results from the current study also showed that the step velocities of the older adults were significantly slower than those of the young adults during both tasks on both the flat and soft surfaces. These results indicate that the step performance of the older adults may have been affected by age-related physiological changes such as sensory degeneration, the extension of central processing, the deterioration of nerve conduction velocity, and muscle atrophy.

Many studies have found that older adults need to dedicate more attentional demand to postural control than young adults and have suggested that the decrease in central processing that occurs with age affects attentional demand [4, 15, 20]. It has also been reported that poor performance in dual tasks could be associated with executive function in older adults. Coppin et al. [16] found that older adults with poor executive function exhibited significantly slower gait speed compared to those with good executive function during the performance of dual tasks. Executive function is defined as the capacity to engage a person in dependent, purposive, and self-serving behavior and can directly affect their strategies for approaching, planning, and carrying out cognitive tasks, and it also plays a role in older adults' ability to adequately allocate attentional resources [16, 21]. We used MΔTMT to assess the difference between the older and the young adults and found that MΔTMT was significantly higher for the older than the young adults, which may confirm that the failure of executive function increases the time needed for central neural processing in older adults.

During the stepping task, the initiation phase depends on the afferent nerve conduction time followed by central neural processing and the efferent nerve conduction time [15]. An interesting finding in our study was that the length of the initiation phase and the %DIP in the dual task were higher for the older than for the young adults when they stepped on the known soft surface, whereas, no significant differences were found between the age groups on the flat surface. We consider that older adults need to dedicate more attentional demand and extend the time required for central nervous processing when stepping on a known soft surface, especially during dual tasks, in order to devise a strategy to avoid losing their postural stability. Brauer et al. [14] also suggested that older adults may prioritize step recovery over cognitive task response, while young adults were able to perform both tasks concurrently. In addition, Bootsma-van der Wiel et al. [20] suggested that older adults may have difficulty in cognitive performance and that some people may prioritize one of the tasks to avoid falling. 

In this study, %DSP was lower and %DDP was higher for the older than for the young adults when stepping on the soft surface, but not on the flat surface. Also, we found that the performances of the older adults were significantly inferior to those of the young adults in the BBT and TUG. From these results, we consider that experience of the older adults had already taught them that the soft surface required more attention before they stepped on it because the reduced amount of afferent information coming from the soles of the feet increased the difficulty of controlling postural stabilization. Therefore, the older adults, who had worse balance than the young adults, chose a strategy in which they reduced the amount of time standing on one leg they spend in the swing phase in order to maintain their stability and adjust their postural control on the soft surface. Perry [22] observed that the onset of the age-related decline in plantar-surface sensitivity occurred in the early part of the seventh decade with a steep increase in vibratory thresholds at 100 Hz. Therefore, we need to expand upon the present study for adults over seventy and attempt to confirm the age-related differences in attentional demand.

 Although not directly comparable, Bowen et al. [23] showed that walking velocity decreased and %DDP (referred to as DST% in the paper) increased in people after a stroke when given a cognitive task and implied that attentional demand affects balance ability in patients with acquired brain damage. In our study, although the older adults had no history of neurological problems, we found that MΔTMT was significantly prolonged in the older adults compared to the young adults; therefore, deterioration of the central nervous system could have an adverse affect on concurrent performance.

As we discussed above, we consider that the attention of older adults has been taken for preparing for posture control when they step onto the soft surface especially in the case of dual task. We conclude that an additional attentional demand could require the extension of step initiation, that is, extra time for central nervous processing as a strategy to avoid losing postural stability, since latent deficits in the processing capacity of the central nervous system in older adults reflect dual task performance [20]. Thus, considering performance not only in dual tasks but also the amount of sensory information from the soles of the feet would contribute to assessing dual task performance in situations similar to those encountered in real life by older adults.

## Figures and Tables

**Table 1:** Participants' characteristics (mean ± SD).

	Young	Old
Height (cm)	157.44 ± 4.39	151.86 ± 3.87**
Max step length (cm)	124.49 ± 2.84	96.19 ± 12.89**
M-TMT-A (sec)	14.91 ± 2.27	38.09 ± 5.96**
M-TMT-B (sec)	29.66 ± 5.96	113.08 ± 51.26**
MΔTMT (sec)	14.74 ± 5.57	74.99 ± 43.83**
BBT (point)	55.9 ± 0.3	54.8 ± 1.4*
TUG (sec)	4.33 ± 0.51	7.23 ± 1.42**

Significant difference among two groups (*P < .05, **P < .01).

**Table 2:** Stepping response parameters compared between ages (mean ± SD).

			Step length (cm)	Step velocity (cm/sec)	Initiation time (sec)	Preparation time (sec)
Flat surface	young	Single	49.99 ± 1.59	53.12 ± 2.48	0.18 ± 0.02	0.26 ± 0.03
		Dual	51.02 ± 1.98	47.10 ± 3.71	0.19 ± 0.03	0.27 ± 0.04
	older	Single	44.21 ± 4.31**	41.05 ± 6.52**	0.21 ± 0.06	0.29 ± 0.06
		Dual	44.61 ± 5.48**	38.13 ± 6.75**	0.22 ± 0.04	0.31 ± 0.04

Soft surface	young	Single	51.14 ± 1.37	54.55 ± 1.24	0.17 ± 0.02	0.25 ± 0.04
		Dual	51.23 ± 2.19	47.40 ± 3.82	0.18 ± 0.02	0.28 ± 0.05
	older	Single	43.62 ± 2.74**	41.92 ± 4.33**	0.20 ± 0.03**	0.27 ± 0.05
		Dual	43.35 ± 5.03**	36.38 ± 7.30**	0.24 ± 0.03**	0.30 ± 0.06

^a^Significant difference from the young adults (**P < .01).

**Table 3:** The mean rate of each phase as a percentage of the total stepping task time compared between ages; DIP = duration of initiation phase, DPP = duration of preparatory phase, DSP = duration of swing phase, and DDP = duration of double-stance phase.

			%DIP	%DPP	%DSP	%DDP
Flat surface	young	Single	18.80 ± 1.88	27.89 ± 2.82	33.84 ± 3.22	19.46 ± 4.81
		Dual	17.75 ± 2.43	25.08 ± 2.86	36.94 ± 2.64	20.23 ± 3.82
	older	Single	19.00 ± 4.08	26.79 ± 3.66	32.07 ± 4.95	22.14 ± 5.35
		Dual	18.94 ± 3.26	25.90 ± 2.54	32.73 ± 4.47*	22.39 ± 6.17

Soft surface	young	Single	17.81 ± 1.54	27.21 ± 3.70	38.29 ± 3.35	16.74 ± 2.71
		Dual	17.03 ± 1.75	25.63 ± 4.19	38.12 ± 3.97	19.22 ± 2.52
	older	Single	19.01 ± 2.82	25.80 ± 3.54	35.06 ± 2.48*	20.09 ± 2.38**
		Dual	19.40 ± 2.46*	23.82 ± 2.62	34.20 ± 3.44*	22.60 ± 4.10*

^a^Significant difference from young adults (*P < .05, **P < .01).
